# Why Children with Severe Bacterial Infection Die: A Population–Based Study of Determinants and Consequences of Suboptimal Care with a Special Emphasis on Methodological Issues

**DOI:** 10.1371/journal.pone.0107286

**Published:** 2014-09-23

**Authors:** Elise Launay, Christèle Gras-Le Guen, Alain Martinot, Rémy Assathiany, Elise Martin, Thomas Blanchais, Catherine Deneux-Tharaux, Jean-Christophe Rozé, Martin Chalumeau

**Affiliations:** 1 CHU Nantes, Hôpital de la Mère et de l′Enfant, Clinique médicale pédiatrique, Faculté de médecine de Nantes, Nantes, France; 2 Inserm U1153, Obstetrical, Perinatal and Pediatric Epidemiology Research Team, Research Center for Epidemiology and Biostatistics Sorbonne Paris Cité (CRESS), Paris Descartes University, Paris, France; 3 CHU Nantes, Hôpital de la Mère et de l′Enfant, Urgences pédiatriques, Faculté de médecine de Nantes, Nantes, France; 4 CHU de Lille, Hôpital R. Salengro, Unité d′urgences pédiatriques et de maladies infectieuses, Université de Lille-Nord de France, Lille, France; 5 Association pour le Recherche et l′Enseignement en Pédiatrie Générale (AREPEGE); Association Française de Pédiatrie Ambulatoire (AFPA), Cabinet de Pédiatrie, Issy-les-Moulineaux, France; 6 CHU Nantes, Hôpital de la Mère et de l′Enfant, Réanimation pédiatrique et néonatale, Faculté de médecine de Nantes, Nantes, France; 7 Hôpital Necker Enfants Malades, AP-HP, Service de pédiatrie générale, Paris Descartes University, Paris, France; Fondazione IRCCS Ca′ Granda Ospedale Maggiore Policlinico, Università degli Studi di Milano, Italy

## Abstract

**Introduction:**

Suboptimal care is frequent in the management of severe bacterial infection. We aimed to evaluate the consequences of suboptimal care in the early management of severe bacterial infection in children and study the determinants.

**Methods:**

A previously reported population-based confidential enquiry included all children (3 months- 16 years) who died of severe bacterial infection in a French area during a 7-year period. Here, we compared the optimality of the management of these cases to that of pediatric patients who survived a severe bacterial infection during the same period for 6 types of care: seeking medical care by parents, evaluation of sepsis signs and detection of severe disease by a physician, timing and dosage of antibiotic therapy, and timing and dosage of saline bolus. Two independent experts blinded to outcome and final diagnosis evaluated the optimality of these care types. The effect of suboptimal care on survival was analyzed by a logistic regression adjusted on confounding factors identified by a causal diagram. Determinants of suboptimal care were analyzed by multivariate multilevel logistic regression.

**Results:**

Suboptimal care was significantly more frequent during early management of the 21 children who died as compared with the 93 survivors: 24% vs 13% (p = 0.003). The most frequent suboptimal care types were delay to seek medical care (20%), under-evaluation of severity by the physician (20%) and delayed antibiotic therapy (24%). Young age (under 1 year) was independently associated with higher risk of suboptimal care, whereas being under the care of a paediatric emergency specialist or a mobile medical unit as compared with a general practitioner was associated with reduced risk.

**Conclusions:**

Suboptimal care in the early management of severe bacterial infection had a global independent negative effect on survival. Suboptimal care may be avoided by better training of primary care physicians in the specifics of pediatric medicine.

## Introduction

Bacterial infection remains a major cause of childhood mortality in industrialised countries. [1] In 2009, Harndern et al. reviewed pediatric deaths in 5 regions of the United Kingdom and found that among the 15% of deaths related to infection, failure to recognise and manage severe bacterial infection (SBI) was the most common avoidable primary care factor. [2] In 2010, we published a population-based study evaluating optimality of care for 21 children who died due to SBI: the initial medical management was suboptimal in 76% of cases, with a delay in seeking medical care in 33%. [3] These alarming frequencies in suboptimal initial care in pediatric patients with SBI do not allow for drawing conclusions on the relationship between suboptimal care and outcomes because both studies focused on patients who died.

The consequences of suboptimal care in pediatric patients with SBI have been examined in 4 studies. [4], [5], [6], [7] All found clinically meaningful and statistically significant associations between suboptimal care and morbidity and mortality. [4], [5], [6], [7] However, the results were limited by methodological concerns such as selection bias related to hospital-based recruitment, [6] classification bias related to arbitrary definition of diagnosis delay as consultation more than once before hospitalisation, [5] non-independent evaluation of the optimality of care, [7] non-justified use of continuous variables in multivariable models, [4], [5], [7] and/or selection of non-appropriate variables for adjustment (without using a causal diagram that could help deal with co-variables that could be confounders or intermediate variables). [8], [9] No study examined the determinants of this suboptimal care to inform corrective actions for parents and healthcare workers.

The aim of the present study was to evaluate the determinants and consequences of suboptimal care in the initial management of SBI in children, using appropriate methodological approaches, to evaluate the relevance of future targeted corrective actions for parents and healthcare workers.

## Methods

### General methodology

The present study is an extension of a previously published population-based confidential enquiry into the quality of initial care in children age from 3 months to 16 years who died of SBI from January 2000 to March 2006 in a geographic zone of France comprising two adjoining administrative districts. [3] The definition of SBI (bacterial infection leading to admission to the pediatric intensive care unit [PICU]), the strategy of identification of cases, and the assessment of exhaustiveness were described in detail in the previous publication. Pediatric care in this area was provided by one university hospital center (in Nantes), four general hospitals, pediatricians in private practice, general practitioners (GPs), and two call centers for medical emergencies that could send emergency mobile medical teams (including physicians specialized in emergency medicine) to the patient's home. For the present study, we defined a control group of pediatric patients who survived a SBI during the same period and in the same geographic region. The organization of care called for all children older than 3 months and requiring hospitalization for SBI to be transferred to the PICU of the Nantes university hospital. Thus, controls were all pediatric patients hospitalised for SBI in the PICU of the hospital during the study period. Controls were identified by discharge codes and the microbiology laboratory electronic files as described previously. [3] The initial research was approved by Institutional Review Committee (Comité de Protection des Personnes Ile de France III) and this extension was approved by the ethics committee of the Nantes university hospital (Groupe Nantais d′Ethique dans le Domaine de la Santé), which approved a waiver of the need for consent. The results were reported according to the STROBE checklist for reporting observational studies. [10]

Data were collected as previously described from the complete patient medical file: a pre-established template reconstructed the timed and dated medical observations with blinding to final diagnosis or outcome. [3] Children whose files were too incomplete to trace the clinical history with sufficient precision were identified and excluded.

### Optimality of care evaluation

Two experts (an experienced pediatrician in private practice and a pediatric intensive care specialist who supervises a pediatric emergency department), blinded to final diagnosis and outcome, independently determined the suboptimal character of the initial management as described previously. [3] These two experts were not involved in the management of any included children. Experts had to justify their final conclusion by giving details on the optimality (optimal or not optimal) of each care in terms of specific criteria selected from national and international clinical practice guidelines applicable during the study period: [11], [12], [13] the timing of administration of antibiotics for meningococcemia (immediate in case of extensive purpura) and the modality of administration of hemodynamic support in septic shock (bolus up to 40 mL/kg in the first hour). As in the study by Nadel *et al.*, [6] which evaluated suboptimal care for meningococcal disease, we defined delay in seeking medical care by parents as the absence of immediate consultation in cases of fever with a purpuric rash or accompanied by other signs of severity: cyanosis, moaning, convulsions, confusion, impairment of higher functions, intense headaches, intense muscle or articular pain, marked asthenia, persistent vomiting, or cold hands or feet. We also arbitrarily considered the failure to seek medical care when a high fever lasted more than 48 hr as a delay in seeking medical care. For each child, we were then able to evaluate the optimality of 6 different key types of care: 1) seeking medical care by parents; 2) evaluation of sepsis signs and detection of severe disease by a physician, 3) timing of antibiotic therapy, 4) dosage of antibiotic therapy, 5) timing of saline bolus, and 6) dosage of saline bolus.

### Analyses

We described the children studied, their demographic characteristics, and their final diagnoses, especially bacteriologic. We analyzed signs of severe disease: signs of sepsis (tachycardia, bradycardia, and tachypnea), [14] presence of tonus disorders, impaired vigilance, respiratory distress, moaning, or other signs of potential SBI, such as meningism or extensive purpura. We described the sequence of care of children (first medical contact and number of consultations before hospitalization).

We assessed the degree of agreement between the two experts for each type of care by calculating the κappa coefficient interpreted with the Landis and Koch scale. [15] In cases of disagreement, the optimal nature of the care was determined by a third expert. We analyzed the 6 categories of suboptimal care by outcome and physicians' qualification. We also analyzed risk factors for death (relation between suboptimal care and death) and determinants of suboptimal medical care (excluding seeking medical care).

**Figure 1:**
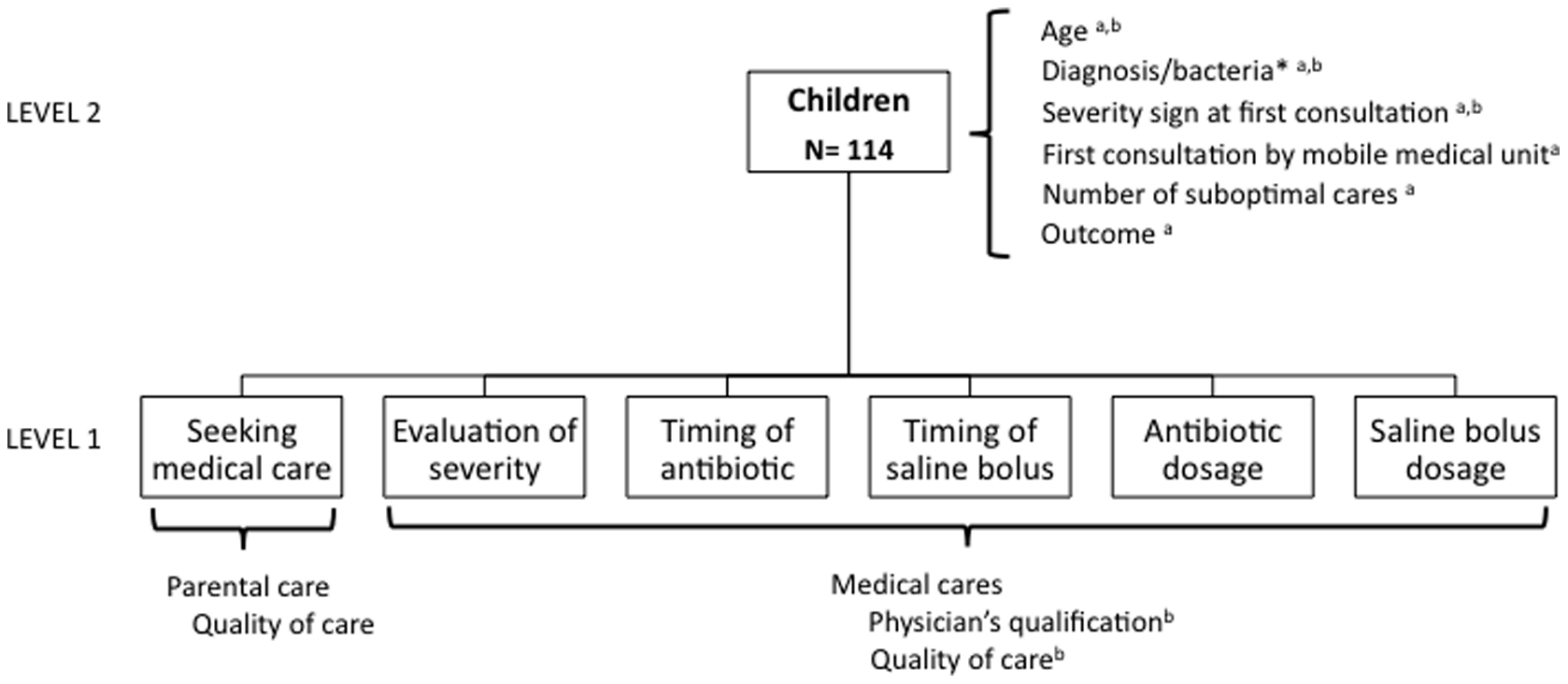
Structure of data. a = co-variables used in the study of the consequences of suboptimal care. b = co-variables used in the study of the determinants of medical sub-optimal care.

We analyzed the crude and adjusted association between number of suboptimal care and death. The number of suboptimal care was the sum of the 6 above-mentioned types for each child and thus ranged from 0 to 6. To identify confounding variables, we built a theorical causal diagram between optimality of care and outcome (dead/alive at discharge from hospital) based on the published pathophysiological concepts of severe sepsis (Figure S1) and adapted from this a “realistic” causal diagram between optimality of initial care (before admission to a PICU) and outcome considering the available data and using DAGitty software (Figure S2). [16], [17] Clinical phenotype was defined by diagnosis (meningitis versus other diagnosis) and two other variables reflecting the measurable intrinsic severity of the disease: presence of severity sign at the first consultation and first consultation by a mobile medical unit (this unit is reserved for patients with the most severe condition in France). Covariables tested on univariate analysis were age of children, diagnosis, sign of severe disease at the first consultation, and first consultation by a mobile medical unit (Figure 1). Relevant variables according to the causal diagram were included in multivariate analyses.

To evaluate the determinants of the quality of initial medical care, we considered each of the 5 medical care types by children. We used a hierarchical regression model that took into account the hierarchical structure of the data (i.e., non-independence of the variables for the 5 care types), and allowed us to include characteristics of care at the care level (level 1; i.e., quality of each care [optimal/suboptimal] and qualification of physician giving the care) and characteristics of the children at the level of the child (level 2; i.e., age of children, diagnosis, and presence of signs of severe disease at first consultation [Figure 1]). We included variables considered associated with suboptimal care (Figure S2). First, we estimated a random intercept model without any variable (“empty” model) to obtain the baseline children-level variance and to test the effect of children. Then, we included care and children characteristics and estimated the association of these variables and quality of care. We calculated the proportion of the model's variance explained by level 1 and level 2 variables defined as (variance of the model with level 1 variables – variance of the empty model)/variance of empty model and (variance of the model with level 1 and level 2 variables – variance of the empty model)/variance of empty model, respectively. Quantitative variables were tested for linearity and transformed into polynomials of the smallest degree when deviation was observed. Analyses involved use of Stata 11 (StataCorp, College Station, TX, USA).

## Results

### Patients and care pathway

In total, 119 patients were eligible; five (4%) were excluded because of incomplete charts, for 114 patients analysed (Figure S3). Overall, 21 children died (18%, 95% confidence interval [95% CI] 11–25) before PICU admission (n = 1) or PICU discharge (n = 20), and 93 survived. The clinical characteristics of the 21 children who died were described elsewhere. [3] The median age of the 114 included children was 2.4 years [interquartile range 0.7–6.7 years], the sex ratio was 1.3 (M/F) and one half had known serious medical conditions at the time of diagnosis (Table 1). More than a half of the children (63%) presented signs of severe disease at the first medical contact. Meningitis was the most frequent diagnosis (57%), followed by purpura fulminans (35%). Meningococcus was found in 47% of cases. The first medical contact was a GP in 66% of cases, an emergency physician in 25%, and a mobile medical unit in 9%; 60% of children were hospitalized after this first medical contact. Children whose first medical contact was the mobile medical unit were more likely to have severity signs at this first medical contact (100% versus 60%, p = 0.01).

**Table 1:** Patient characteristics and care pathways before admission to a pediatric intensive care unit, quality of care and their association with outcome by dead and alive children and univariate and multivariate analysis.

	Total n = 114 (%)	Dead n = 21 (%)	Alive n = 93 (%)	Univariate analysis			Multivariate analysis§		
				OR	95% CI	p	aOR	95% CI	p
**PATIENTS**									
**Age, yr**									
Median^a^ [IQR]	2.4 [0.7–6.7]	2.0 [0.9–2.8]	2.9 [0.7–7.1]	0.85	0.73–1.0	0.057	0.82	0.68–0.99	0.04
<1 yr	32 (28)	7 (33)	25 (27)						
1 to 2 yr	21 (18)	4 (19)	17 (18)			0.01			
2 to 5 yr	27 (24)	9 (43)	18 (19)						
≥5 yr	34 (30)	1 (5)	33 (36)						
**Sex ratio M/F**	1.28	1.33	1.27			0.92			
**Underlying medical conditions,** n (%)	39 (52)	7 (33)	32 (34)			0.93			
**Severity signs at first medical contact** b, n (%)	72 (63)	16 (76)	56 (60)			0.17			
**Final diagnosis,** n (%)									
Purpura fulminans and others^c^	49 (43)	14 (67)	35 (38)	1	-	-	1	-	-
Meningitis	65 (57)	7 (33)	58 (62)	0.30	0.11–0.85	0.02	0.31	0.10–0.98	0.047
**Bacteria involved**, n (%)									
*Streptococcus pneumoniae*	31 (27)	3 (14)	28 (30)						
*Neisseria meningitidis*	54 (47)	12 (57)	42 (45)						
Other^d^	11 (10)	4 (19)	7 (8)			0.17			
No documentation	18 (16)	2 (10)	16 (17)						
with purpura fulminans	10 (55)	1 (50)	9 (56)						
**CARE PATHWAYS**									
**First medical contact**									
GP or emergency physician, n (%)	104 (91)	15 (71)	89 (96)	1	-	-	1	-	-
Mobile medical unit	10 (9)	6 (29)	4 (4)	8.9	2.05–38.6	<0.001	8.72	1.76–43.28	0.008
**No of medical contacts,** n (%)									
1	68 (60)	15 (71)	53 (57)						
2	37 (32)	6 (29)	31 (33)			0.43			
>2	9 (8)	0	9 (10)						
**QUALITY OF CARES**									
**No. of suboptimal care, by children**									
Median [IQR]^a^	1 [0–1]	1 [0–2]	0 [0–1]	1.55	1.06–2.26	0.025	1.65	1.07–2.54	0.022
0	55 (48)	7 (33)	48 (52)						
1	31 (27)	5 (24)	26 (28)						
2	17 (15)	5 (24)	12 (13)						
3	5 (4)	1 (5)	4 (4)						
4	6 (5)	3 (14)	3 (3)						
**No suboptimal care/no care, % [95% CI]**	15 [12]–[18]	24 [16]–[32]	13 [10]–[16]			0.003			
**Care types**									
**Parental care,** n (%)									
***Seeking medical care***									
Suboptimal	23 (20)	6 (29)	17 (18)	1.79	0.60–5.34	0.29			
Optimal	91 (80)	15 (71)	76 (82)	1	-	-			
**Medical care,** n (%)									
***Evaluation of severity***									
Suboptimal	23 (20)	7 (33)	16 (17)	2.40	0.82–7.03	0.10			
Optimal	91 (80)	14 (67)	77 (83)	1	-	-			
***Antibiotic therapy timing***									
Suboptimal	27 (24)	5 (24)	22 (24)	1	0.33–3.08	0.98			
Optimal	87 (76)	16 (76)	71 (76)	1	-	-			
***Saline bolus timing***									
Suboptimal	14 (12)	5 (24)	9 (10)	2.92	0.84–10.1	0.08			
Optimal	100 (88)	16 (76)	84 (90)	1	-	-			
***Saline bolus dosage***									
Suboptimal	12 (11)	7 (33)	5 (5)	**8.80**	2.23–34.7	0.002			
Optimal	102 (89)	14 (67)	88 (95)	1	-	-			
***Antibiotic therapy dosage***									
Suboptimal	5 (4)	0 (0)	5 (5)	0.67	0.01–6.03	0.29			
Optimal	109 (96)	21 (100)	88 (95)	1	-	-			

aOR, adjusted odds ratio; 95% CI, 95% confidence interval; IQR, interquartile range.

§ Logistic regression model.

a Age and no. of suboptimal care were treated as continuous variables (no deviation to linearity).

b Severity signs were hemodynamic failure, purpura, conscientiousness impairment, respiratory distress, meningism, behavioural changes or hypotonia.

c Others were 2 pneumonia with pleural effusion and a septic shock following pyelonephritis in a child with malformative uropathy in the deceased group, and 2 septic shock on bacterial cellulitis and a bacterial tracheitis in the survivor group.

d Others were, for survivors, *Haemophilus influenzae* (n = 3), Group B *Streptococcus* (n = 1), *Staphylococcus aureus* (n = 1), and for deceased children, *E.coli* (n = 1), Group A *Streptococcus* (n = 1), *Salmonella spp (n = 1)* and *Mycoplama pneumoniae* (n = 1).

### Optimality of care

Agreement between experts was “moderate” for evaluation of the optimality of the delay to seek medical care and for saline bolus dosage, with a κ coefficient of 0.40±0.06 and 0.46±0.09, respectively (p<0.001). Agreement with the optimality of the 4 other medical care types (severity evaluation, antibiotic therapy timing and dosage, and saline bolus timing) was “substantial” or “almost perfect,” with κ 0.78±0.09; 0.78±0.09; 0.67±0.09 and 0.88±0.09, respectively (p<0.001). Overall, 52% of children received at least one care type evaluated as suboptimal, and 25% received two or more suboptimal care types (Table 1). Among the 684 individual care types delivered, 104 (15%, 95% CI 12–18%) were suboptimal. Parental delay in seeking medical care and physician underestimation of severity and delayed antibiotic administration accounted for 70% of this suboptimal care (22%, 22% and 26%, respectively). The frequency of suboptimal care in the initial management did not significantly decrease over the years (19% to 15% from 2000 to 2005; p for trend>0.8) nor did the frequency of each type of care (p>0.2).

### Factors associated with outcome

As compared with children who died, survivors were more frequently older than 5 years (p<0.05; Table 1) and diagnosed as having meningitis (62% vs 33%). The two groups did not differ in other demographic, clinical or bacteriologic characteristics or total number of medical contacts before admission to the ICU (p>0.1, Table 1). For children who died, the first medical contact was frequently a mobile medical unit (vs GP office or hospital emergency department): 29% vs 4% (p<0.001). The proportion of suboptimal care among all care types during the initial management was higher for children who died than survived: 24% vs 13% (95% CI of the risk difference: 9–13%). On univariate analysis, insufficient saline bolus was significantly associated with death (OR = 8.8; 95% CI: 2.23–34.7), under-evaluation of severity and delay to administer saline bolus was associated but not significantly with death (OR = 2.74; 95% CI: 0.82–7.03 and 2.92; 95% CI: 0.84–10.1 respectively) (Table 1).

After adjustment for confounders, each suboptimal care (continuous variable, no deviance to linearity) increased the odds of death by 65% (adjusted odds ratio [aOR] 1.65, 95% CI 1.08–2.54, p = 0.02) (Table 1). Each year of age (continuous variable, no deviation to linearity) decreased the odds of death (aOR 0.82, 95% CI 0.68–0.99, p = 0.04), as did having meningitis as compared with other diagnoses (aOR 0.31, 95% CI 0.10–0.98, p = 0.047). A first medical contact by the mobile medical unit was associated with an adverse outcome (aOR 8.72, 95% CI 1.76–43.28, p = 0.008) (Table 1).

### Determinants of optimality of medical care

Among the 570 cares received by children during their initial management (Table 2), the repartition of suboptimal care differed by physician qualification. The proportion of suboptimal care was 33% for those provided by a GP, 30% for those in adult emergency settings, 24% for those in pediatric wards, 10% for those in pediatric emergency care and 7% for those in the mobile medical unit (p<0.001). The proportion of under-evaluation of severity was 30% for a GP, 9% for pediatric emergency care and 0% for the mobile medical unit (p = 0.001). The proportion of delayed antibiotic therapy was 50% for a GP, 20% for pediatric emergency care and 0% for the mobile medical unit (p = 0.02). The other types of care (antibiotic therapy dosage and timing and dosage of saline bolus) did not differ by physician qualification.

**Table 2:** Risk factors for medical suboptimal care.

	Optimal n = 489 (%)	Suboptimal n = 81 (%)	Univariate analysis			Multivariate analysis *,**		
			OR	95% CI	p	aOR	95% CI	p
**Age**								
<1 yr	125 (26)	35 (43)	1			1		
1–2	95 (19)	10 (12)	0.38	0.18–0.81	0.009	0.32	0.11–0.98	0.046
2–5 yr	119 (24)	16 (20)	0.48	0.25–0.92	0.02	0.37	0.14–0.98	0.045
≥5 yr	150 (31)	20 (25)	0.48	0.26–0.87	0.01	0.24	0.09–0.64	0.004
**Physician qualification**, n (%)								
General practitioner	55 (11)	27 (33)	1			1		
Adult emergency	16 (3)	7 (9)	0.90	0.33–2.44	0.82	0.63	0.15–2.62	0.53
Pediatric emergency	322 (66)	37 (46)	0.23	0.13–0.42	<0.001	0.16	0.08–0.35	<0.001
Mobile medical unit	83 (17)	6 (7)	0.15	0.05–0.40	<0.001	0.09	0.03–0.31	<0.001
Pediatric ward	13 (3)	4 (5)	0.63	0.18–2.13	0.45	0.65	0.11–3.67	0.63
**Severity signs at first consultation**, n (%)								
No	182 (87)	28 (13)	1			1		
Yes	307 (85)	53(15)	1.12	0.68–1.84	0.6	1.3	0.59–2.90	0.51
**Final diagnosis**, n (%)								
Other	210 (86)	35 (14)	1			1		
Meningitis	279 (86)	46 (14)	0.99	0.62–1.59	0.9	0.73	0.34–1.59	0.43

*Multivariate analysis involved a hierarchical logistic regression model with random intercept and effects.

**Significant associations remained when age was transformed into polynomials (X = 10/[age – 2.5]), aOR for age 1.04, 95% CI 1.01–1.07, p = 0.003.

On univariate analysis, younger children (<1 year) were at increased risk of suboptimal care (see Table 2), and odds of suboptimal care were lower with pediatric emergency or mobile medical unit care than GP care (OR 0.23, 95% CI 0.13–0.42; and OR 0.15, 95% CI 0.05–0.40, respectively) (Table 2). We found no association between final diagnosis or presence of severity sign at first medical contact and quality of care. The optimality of care varied significantly between children (i.e., children effect, empty model, p<0.001). After adjustment in a multilevel multivariate model, the association between optimality of care and age of children (dichotomised in 4 classes or transformed in polynomials) and physician qualification remained stable (Table 2). The variance of the empty model was 1.56, that of the model with a level 1 variable (physician qualification) was 1.54 and that of the full model (level 1 and 2 variables) was 1.21. Level 1 variables explained 2% of the variance, whereas level 1 and 2 variables explained 21% of the variance.

## Discussion

We found a strong association of suboptimal medical care and death for children with an SBI: each suboptimal care increased the odds of death by 65%. Some types of care, particularly the dosage of saline bolus, were associated more with death than others. The gold standard to demonstrate causality in medical research is a controlled double blind randomised trial, but such studies are obviously not ethical in the case of SBI. Observational study analysis of causal association requires being aware of the risk of bias. [18] Here, we studied the determinants and consequences of suboptimal care in the early management of SBI in pediatric patients using an adequate approach to deal with the structure and type of data, including multilevel analysis and fractional polynomials [19], [20], [21] while minimizing the selection bias for children who died by using a population recruitment pattern with exhaustivity checking. As recommended by methodological standards, [22] suboptimality of care was evaluated by two independent experts who were blinded to the final diagnosis and outcome, with an overall high level of agreement between experts.

The strength of the significant association between suboptimal care and death remained nearly unchanged after adjustment for potential confounders: age of children, final diagnosis and initial severity of disease (represented as having a first medical contact by a mobile medical unit). However, some variables were inaccurately measured and/or some explanatory variables were lacking in the model. Indeed, we show a gap between the number of variables in the theoretical causal diagram and the one used (Figure S1 and Figure S2). For example, we could have explained more accurately the risk of death by considering genetic susceptibility to infection or bacterial virulence. [23], [24] We were also limited in the evaluation of initial severity of the infection because of the retrospective design of the study. First medical contact by a mobile medical unit is an objective and reliable evaluation of clinical severity because in France, the mobile medical unit aims to care for children with the most severe disease who could not be transported to the hospital before receiving emergency care. Nevertheless, the presence of a severity sign at the first medical examination is a more arguable reflection of intrinsic severity because of the retrospective design of the study. For example, data on vital signs at the first medical contact, which are a key point to evaluate clinical severity in children in the context of SBI, [25] were sometimes missing, which led to inaccurate evaluation of severity of the disease for statistical analysis and also difficulty for the expert to accurately assess the optimality of the severity evaluation. Experts evaluated only misinterpretation of vital signs when they were mentioned. Here, we demonstrated the significant and independent global effect of suboptimal care on outcome, but we cannot affirm that suboptimal care in the early management was directly responsible for death because suboptimal care in the PICU was not assessed. Moreover, we analyzed only six types of care for each management and not all types. We could not examine the time effect and the total number of care. The total number of care could be the result of intrinsic severity (severely ill children requiring more care and sometimes showing a fulminant evolution) or the result of previous suboptimal care (inadequate care could lead to worsened disease, which then requires more care). The time effect could have been considered in a marginal structural model (Figure S1), but such a model requires timely detailed information that cannot be obtained with a retrospective study. [26]

We did not include children with SBI who survived but were not hospitalized in a PICU. It could be argued that we over-evaluated the frequency of suboptimal care because children with SBI admitted to a PICU may have received more suboptimal care, which caused clinical worsening and then admission to a PICU as compared with children who received adequate care and would not have required admission to a PICU. Thus, this selection bias could have led to an under-evaluation of the association of suboptimal care and death because these children not hospitalized in a PICU and having received potentially more optimal care would most probably have survived.

The generalization of our results may be limited because the bacterial epidemiology may have changed since the study period. In France, conjugate vaccines against *Haemophilus influenzae, Neisseria meningitidis C and Streptococcus pneumoniae* with 7 and 13 valences were routinely recommended for all children by health authorities in 1992, 2009, 2002 and 2009, respectively. Invasive infection due to *H. influenzae* had almost disappeared during the study period. Reported cases of invasive pneumococcal infection decreased after vaccination introduction for only children younger than 2 years old. Incidences were 29 per 100 000 in 2001, 25 per 100 000 in 2004 and 18 per 100 000 in 2012 (meningitis and bacteremia). No significant changes were observed for older children. [27] Vaccine against meningococcus C had a too low coverage in the pediatric population to evidence a decrease in invasive infection due to *N. meningitidis* C since study period. [28] Thus, since the study period, the pattern of SBI may have changed for invasive pneumococcal infection in children less than 2 years old.

We did not observe a significant decrease in suboptimality of care across the years even though French recommendations concerning immediate administration of antibiotic therapy with purpura fuminans were largely diffused in 2000 and the Surviving Sepsis campaign began in 2003. [29], [30] This finding highlights that simple diffusion of written recommendations are not enough to quickly modify practices of a large healthcare professional public. [31]

The analysis of suboptimal care determinants allowed us to identify potential targets for corrective actions. Young age (<1 year) was independently associated with increased risk of suboptimal care, whereas being under the care of a paediatric emergency specialist or a mobile medical unit physician was associated with reduced risk. Similar conclusions were reached by Dhamar *et al.* in a retrospective review of the quality of care received by 304 children with serious illnesses receiving treatment in 5 emergency departments in California between 2000 and 2003: after adjustment for confounding factors with a hierarchical model, younger children were at increased risk of receiving suboptimal care, and quality of care was better when provided by pediatric emergency physicians as compared with a GP. [32] Young age of children also appeared to be a barrier to optimal management of critical illness in community hospitals according to a qualitative study. [33] Corrective actions should then target the GP, in training and established, and focus on clinical evaluation of the youngest children.

Seeking medical care was considered delayed in 20% of our cases and accounted for 22% of the suboptimal care. We could not study the determinants of this delay, but French parents were previously found to poorly recognise purpuric rash. [34] Nevertheless, methods to recognize purpuric rash are warranted for not missing severe bacterial infections. [35] Parents worrying about their child's health has also been identified as a good marker of severe infection, although this sign is often missing, even with severe infection. [36] The better understanding of why parents are worried or not could be helpful to optimize early detection of sepsis.

## Conclusions

Thanks to an adequate strategy for data analysis, we showed a significant association of suboptimal care for children with SBI and death. We identified determinants that could be acted on to optimize early management of SBI in children and then hopefully reduce the incidence of death. Physicians who are in charge of febrile children should pay particular attention to children younger than 1 year and systematically evaluate vital signs (pulse, respiratory rate, consciousness, capillary refill) that allow for early recognition of severe sepsis. Physicians and parents could be warned via widely distributed flyers or even television, as has been efficient in United Kindom by the meningitis research foundation. [37] Physicians who rarely experience vital emergency situations could also benefit from a simulated training program. [38]

## Supporting Information

Figure S1 **Theoretical causal diagrams between optimality of care and death reflecting time-dependance of exposure and confounding factors.** a: summarized diagram with C representing confounding factors; E, exposition (optimality of care); F, risk factors for exposition (determinants of optimality); and Y, outcome (survival status). b: more complete diagram with H representing host factors (age, genetic and non-genetic susceptibility to infection); B, bacterial factors (type of infection, bacterial specie/serotype, virulence, inoculum); O, optimality of care; S, clinical severity; P, physician characteristics (qualification, clinical experience etc.); Pa, parent characteristics (educational/socioeconomic status, facility of access to health care systems etc.). Indices represent different time points (from 0 to k) (Inspired by Robins et al, Epidemiology, September 2000, Vol. 11 No. 5).
(TIF)

Figure S2 **“Realistic” causal diagram between optimality of care before admission to a pediatric intensive care unit (PICU) and death.** This diagram was established with DAGitty considering available variables. [16] The green circle with triangle inside represents exposure; blue circle with stick inside, outcome; green circles, exposure ancestors; pink circles, confounding factors; pink vectors, biasing pathway; green vectors, causal pathways; grey vectors, ancestor pathway. Clinical phenotype was represented by final diagnosis, severity signs at the first medical contact and first medical contact by a medical mobile unit.
(TIF)

Figure S3 **Study flowchart.**
(TIF)
