# Supplementary figures and images for: Why Children with Severe Bacterial Infection Die: A Population–Based Study of Determinants and Consequences of Suboptimal Care with a Special Emphasis on Methodological Issues
Source: PLoS One. 2014 Sep 23;9(9):e107286. doi: 10.1371/journal.pone.0107286 (PMC4172434; doi:10.1371/journal.pone.0107286)

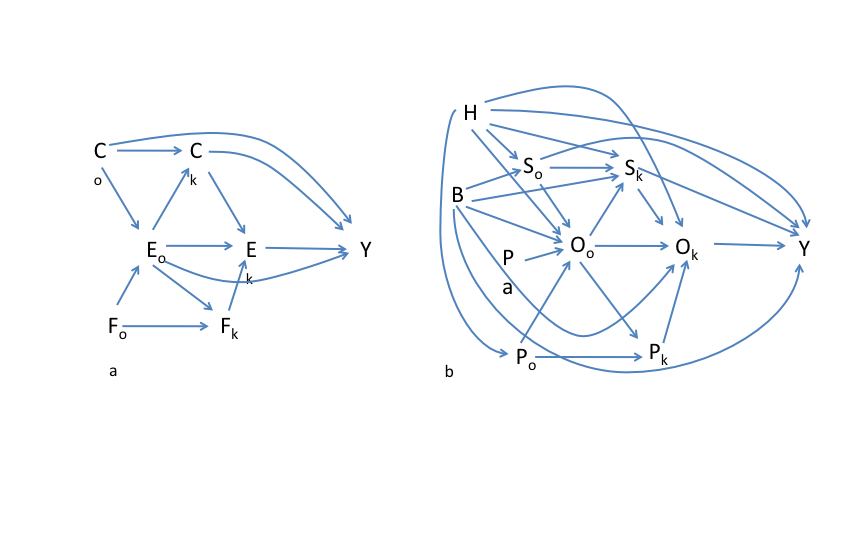

Supplement: Figure S1 — Theoretical causal diagrams between optimality of care and death reflecting time-dependance of exposure and confounding factors. a: summarized diagram with C representing confounding factors; E, exposition (optimality of care); F, risk factors for exposition (determinants of optimality); and Y, outcome (survival status). b: more complete diagram with H representing host factors (age, genetic and non-genetic susceptibility to infection); B, bacterial factors (type of infection, bacterial specie/serotype, virulence, inoculum); O, optimality of care; S, clinical severity; P, physician characteristics (qualification, clinical experience etc.); Pa, parent characteristics (educational/socioeconomic status, facility of access to health care systems etc.). Indices represent different time points (from 0 to k) (Inspired by Robins et al, Epidemiology, September 2000, Vol. 11 No. 5). (TIF) [file pone.0107286.s001.tif]

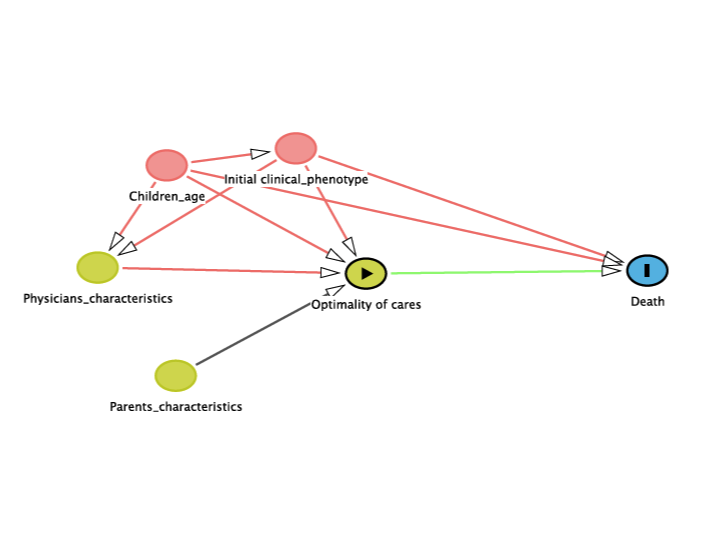

Supplement: Figure S2 — “Realistic” causal diagram between optimality of care before admission to a pediatric intensive care unit (PICU) and death. This diagram was established with DAGitty considering available variables. [16] The green circle with triangle inside represents exposure; blue circle with stick inside, outcome; green circles, exposure ancestors; pink circles, confounding factors; pink vectors, biasing pathway; green vectors, causal pathways; grey vectors, ancestor pathway. Clinical phenotype was represented by final diagnosis, severity signs at the first medical contact and first medical contact by a medical mobile unit. (TIF) [file pone.0107286.s002.tif]
